# Differential biomarker responses of hemolysis, inflammation, and myocardial injury after pulsed-field pulmonary vein isolation: balloon-in-basket vs. circular catheter systems

**DOI:** 10.3389/fcvm.2026.1774443

**Published:** 2026-05-29

**Authors:** Jan-Per Wenzel, Raed Abdessadok, Charlotte Eitel, Sorin Popescu, Suzanne de Waha, Tanja Zeller, Karl-Heinz Kuck, Roland Richard Tilz, Sascha Hatahet

**Affiliations:** 1 Department of Rhythmology, Heart Center Lübeck, University Hospital Schleswig-Holstein, Lübeck, Germany; 2 German Center for Cardiovascular Research (DZHK), Partner Site, Lübeck, Germany; 3 Institute for Cardiogenetics, University Hospital Schleswig-Holstein, Lübeck, Germany

**Keywords:** atrial fibrillation, ballon-in-basket, circular catheter, hemolysis, inflammation, myocardial injury, pulmonary vein isolation, pulsed-field ablation

## Abstract

**Background:**

Pulsed-field ablation (PFA) is a non-thermal energy source for pulmonary vein isolation (PVI). Data comparing the biological impact of different PFA technologies remain limited. This study assessed changes in biomarkers of hemolysis, inflammation, and biochemical myocardial injury using two PFA systems: a balloon-in-basket (BiB) and a circular catheter (PS).

**Methods:**

This prospective, single-center, non-randomized study enrolled consecutive patients undergoing first-time PVI. leukocytes, C-reactive protein (CRP), platelets, lactate dehydrogenase (LDH), haptoglobin, total bilirubin, troponin T, creatine kinase (CK), myoglobin, creatinine, and estimated glomerular filtration rate (eGFR) were before and 16–18 h after ablation.

**Results:**

Forty patients were included (BiB *n* = 20, PS *n* = 20). Baseline characteristics were comparable. Acute PVI was achieved in all cases. BiB required fewer applications (16 vs. 32, *p* < 0.001), more contrast (45 vs. 30 mL, *p* < 0.001), and had longer procedure and LA dwell times (*p* < 0.001). Both systems caused significant increases in leukocytes, CRP, LDH, troponin, and CK without inter-group differences. Haptoglobin decreased significantly in both, more pronounced with PS (Δ −13 vs. −4 mg/dL, *p* = 0.090). Renal function remained stable overall, with one PS patient developing an acute kidney injury. In the BiB group, Δ-Hemoglobin correlated inversely with application number (*p* = 0.046). In the PS group, *Δ*-Troponin and *Δ*-CK correlated positively (*p* < 0.05).

**Conclusion:**

Despite comparable overall biomarker responses, a numerically greater, non-significant haptoglobin decline and an isolated, likely multifactorial transient AKI in the PS group may suggest differences in biomarker responses, warranting further investigation in larger studies. However, the clinical relevance of these findings remains uncertain.

## GRAPHICAL ABSTRACT

**Figure:**
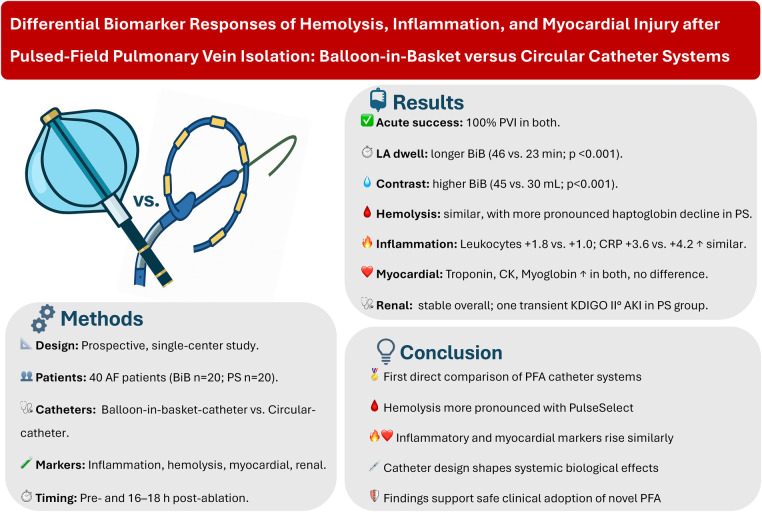

## What's new?

- Ballon-in-basket and circular PFA catheters were associated with increases in inflammatory and biochemical myocardial injury.
- Haptoglobin decreased in both groups, more pronounced with PulseSelect.
- Renal function remained stable in most patients in both groups, with one PS patient developing a transient AKI representing a single, likely multifactorial observation.
- Overall Biomarker responses were comparable, with modest system-specific differences.

## Introduction

Pulsed-field ablation (PFA) has emerged as an innovative approach in atrial fibrillation (AF) therapy, providing a non-thermal method for pulmonary vein isolation. Using high-voltage electric pulses, PFA induces irreversible electroporation of cardiomyocytes, thereby creating durable lesions while minimizing collateral injury to surrounding structures. Recent multicenter studies have confirmed its high procedural efficacy and superior safety profile when compared to thermal energy sources (1–3).

Beyond lesion formation, PFA induces systemic effects, most notably intravascular hemolysis, characterized by increased LDH and bilirubin with reduced haptoglobin, and in rare cases progression to pigment-induced acute kidney injury (2). In addition, elevations in inflammatory and biochemical myocardial injury, including C-reactive protein (CRP), leukocytes, troponin, and creatine kinase (CK), have been reported after ablation. While CRP has been linked to atrial remodeling and arrhythmia recurrence (4–7), troponin and CK might primarily reflect the extent of myocardial damage, though their prognostic value for long-term outcomes remains debated.

Among PFA technologies, the VOLT™ balloon-in-basket catheter (BiB; Abbott) represents a novel approach, characterized by its balloon-in-basket configuration, distinct electrode arrangement, and balloon-mediated energy dispersion (3). In contrast, the PulseSelect^TM^ PFA catheter (PS; Medtronic) is a multipolar loop-based system advanced over a guidewire that delivers energy via a biphasic pulse waveform (8). Such differences in design and energy application may modulate systemic responses.

**Figure 1:**
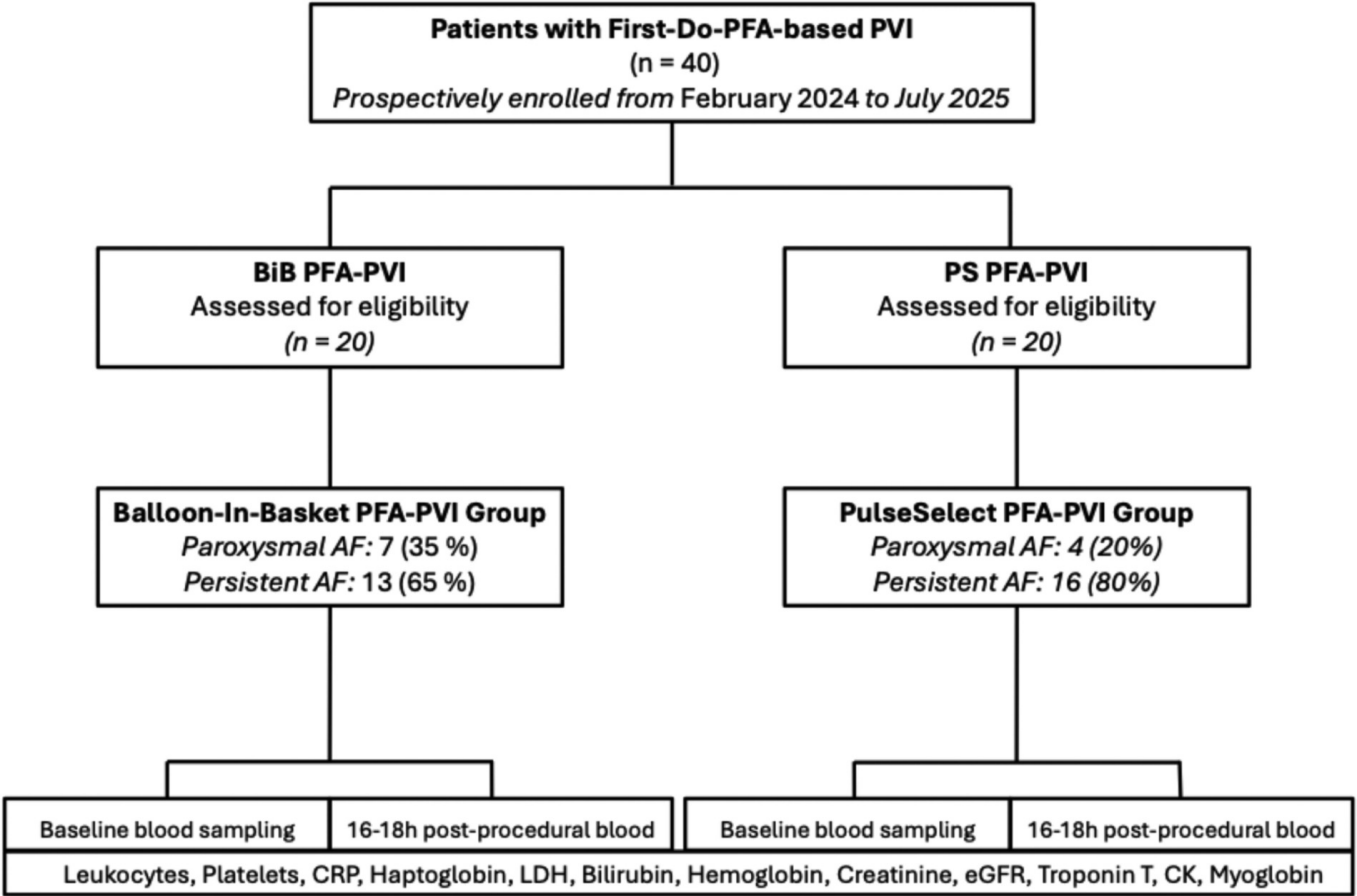
Study PRISMA (STROBE format). BiB, Balloon-in-basket; PS, PulseSelect; AF, atrial fibrillation; PFA, pulsed field ablation; PVI, pulmonary vein isolation, CK, Creatine kinase; CRP, C-reactive protein; eGFR, Estimated glomerular filtration rate.

To date, no studies have directly compared the systemic effects of the recently released BiB (limited market release in Europe) catheter and the circular PS catheter with respect to hemolysis, inflammation, and myocardial injury. This study therefore aimed to characterize and contrast acute biomarker responses between both platforms. To our knowledge, this represents the first comparative analysis of biomarker responses between these two PFA systems. The study design is shown in Figure 1.

## Methods

### Study population and trial design

This prospective, single-center, non-randomized study investigated systemic biomarker changes after PFA-based PVI using two catheter platforms: the PS and the BiB catheter. From February 2024 to July 2025, consecutive patients with paroxysmal or persistent AF were enrolled. All procedures were carried out at the Heart Center Lübeck and documented within the institutional ablation registry.

Inclusion criteria were age ≥18 years, documented AF, and written informed consent. Patients with active infection, autoimmune or chronic inflammatory disease, recent myocardial infarction, neuromuscular disorders, or severe hepatic dysfunction were excluded to avoid biomarker confounding. The study was approved by the institutional ethics committee (Lübeck Ablation Registry, WF-028/15) and conducted in accordance with the Declaration of Helsinki.

### General procedural management

All patients underwent standardized preprocedural evaluation according to institutional protocols. In those with elevated thromboembolic risk, transesophageal echocardiography was performed to rule out intracardiac thrombus. Anticoagulation with vitamin K antagonists was maintained at a therapeutic INR (2.0–3.0), while direct oral anticoagulants (DOACs) were withheld on the morning of the procedure. Ablation was conducted under deep sedation using propofol, midazolam, and fentanyl. In selected cases, continuous propofol infusion was omitted to preserve patient responsiveness, and a multimodal analgesic regimen consisting of metamizole, midazolam, fentanyl, and lidocaine was applied.

Femoral venous access was obtained via one or two ultrasound-guided punctures with 8 Fr sheaths, followed by placement of a diagnostic catheter in the coronary sinus.

Transseptal puncture was performed under fluoroscopic guidance using the modified Brockenbrough technique. After entry into the left atrium, intravenous unfractionated heparin was administered to maintain an activated clotting time >300 s, and left atrial access was secured with an SL1 sheath (Abbott). The catheter system used in each case was determined by device availability during the study period, reflecting routine clinical practice. Both systems were used within an overlapping time frame, with temporal variability in availability. The PS system was available from June to July 2025, whereas the BiB system was available from February 2024 through July 2025.

### Circular PFA -catheter procedure

The PS catheter (9F) was advanced into the left atrium over a guidewire following transseptal puncture. The catheter delivers a controlled biphasic, bipolar waveform via a circular array of nine 3 mm gold electrodes. Secure wall contact was achieved by advancing the circular array to the pulmonary vein (PV) ostium, where fluoroscopy was used to assess changes in catheter angulation upon contact with the PV wall.

Each application consisted of four biphasic, bipolar pulse trains lasting 100–200 ms. After each application, the catheter was rotated in a systematic sequence to achieve full circumferential lesion sets: starting at the roof position, followed by 45° clockwise or counterclockwise rotations to target the anterior, inferior, and posterior PV aspects. Initially, four applications were delivered at the ostium, followed by four additional applications at the antral level. For the posterior aspect, sheath manipulation involved clockwise torque for left-sided PVs and counterclockwise torque for right-sided PVs. Conversely, for the anterior aspect, the sheath was turned counterclockwise for left-sided PVs and clockwise for right-sided PVs.

### Balloon-in-basket PFA-catheter procedure

The catheter was introduced into the left atrium via a steerable 13 Fr Agilis^TM^ NxT sheath (Abbott) over a 0.035 inch guidewire. The expandable balloon incorporates eight active spline electrodes, enabling single-shot circumferential PFA delivery. Additionally, 9 of the 20 patients treated with the BiB system were co-enrolled in the VOLT CE Mark study, sponsored by Abbott. For those, pre-ablation three-dimensional voltage mapping of the left atrium was carried out using a high-density mapping catheter. Pulses were applied at 1,800 V with at least two rotated applications per vein, or at 1,400 V with typically three applications, with a maximum of four applications per vein. Phrenic nerve pacing was routinely performed during right-sided PV ablation. In cases with diaphragmatic capture, ablation was performed at 1,400 V with ≥3 applications. The integrated contact indicator was used to verify secure PV wall contact during ablation. Electroanatomical mapping was conducted using the EnsiteX system (Abbott), with the BiB catheter employed for map acquisition after PVI. For patients being enrolled in VOLT CE Mark study, remap was performed after a 20 min waiting period. Acute procedural success was confirmed by demonstration of entrance block in all targeted PVs.

### Postprocedural management

Hemostasis was achieved using either vascular closure devices or figure-of-eight sutures combined with compression bandage. Compression bandages were removed after 1–4 h, whereas sutures were removed on the following day. Transthoracic echocardiography was routinely performed 1 h post-procedure and again on the first postoperative day to rule out pericardial effusion. Oral anticoagulation was restarted 6 h after ablation and continued for a minimum of two months. Long-term anticoagulation was guided by individual thromboembolic risk (CHA₂DS₂-VA score) in accordance with current guideline recommendations.

### Blood sampling and analysis

Venous blood samples were obtained at two predefined time points: (1) after femoral venous access and prior to ablation, and (2) on the morning of the first postoperative day. All samples were collected in the fasting state. In one PS patient who showed marked changes in renal parameters, an additional blood sample was obtained the following morning. The biomarker panel included leukocytes, platelets, C-reactive protein (CRP), high-sensitivity troponin T, creatine kinase (CK), CK-MB, myoglobin, serum creatinine, and estimated glomerular filtration rate (eGFR).

Leukocytes and platelets were measured in EDTA blood by fluorescence flow cytometry (Sysmex XN-9000). CRP was determined by immunoturbidimetry (Cobas c503). Troponin T and myoglobin were quantified using electrochemiluminescence immunoassay (Cobas 801). CK and CK-MB were measured enzymatically by UV photometry (Cobas c702), creatinine by Cobas c503, and eGFR was calculated using the CKD-EPI formula. CK-MB was analyzed only in patients with elevated baseline values. All laboratory analyses were performed at the central laboratory of the University Hospital Schleswig-Holstein in compliance with certified, validated protocols.

### Statistical analysis

Continuous data were tested for normality using the Shapiro–Wilk test and are presented as mean ± SD or median (Q1, Q3), as appropriate. Between-group comparisons were performed using independent-samples *t*-tests or Mann–Whitney *U*-tests. Within-group comparisons used paired *t*-tests or Wilcoxon signed-rank tests. Correlations between biomarker changes and ablation parameters were analyzed with Pearson or Spearman coefficients, depending on distribution. Categorical variables are reported as absolute and relative frequencies and compared using Fisher's exact test. No correction for multiple comparisons was performed, and findings should be interpreted in an exploratory context. Analyses were conducted using IBM SPSS Statistics v29.0.1.0 (IBM Corp., Armonk, NY, USA). A two-sided *p*-value < 0.05 was considered statistically significant.

## Results

### Baseline characteristics

A total of 40 patients were included, 20 treated with the BiB catheter and 20 with the PS catheter. Baseline characteristics were largely comparable between groups. Patients in the PS group were slightly older (73 ± 9 vs. 71 ± 8.6 years, *p* = 0.593) with similar proportions of persistent AF (80% vs. 65%, *p* = 0.480) and paroxysmal AF (20% vs. 35%, *p* = 0.480). Comorbidities such as hypertension (85% vs. 80%), diabetes (15% vs. 20%), coronary artery disease (15% vs. 25%), and heart failure (30% vs. 15%) showed no significant differences. Left ventricular function, atrial dimensions, BMI, and CHA_2_DS_2_-VA score were comparable between groups. Overall, both cohorts exhibited balanced baseline characteristics (Table 1).

**Table 1:** Baseline characteristics of the study population.

Variable	BiB-catheter Group (*n* = 20)	PS-catheter Group (*n* = 20)	*p*-value
Female sex, *n* (%)	8 (40%)	10 (50%)	0.751
Age (years)	71 ± 8.6	73 ± 9.0	0.593
Paroxysmal AF, *n* (%)	7 (35%)	4 (20%)	0.480
Arterial hypertension, *n* (%)	16 (80%)	17 (85%)	1.000
Diabetes, *n* (%)	4 (20%)	3 (15%)	1.000
Coronary artery disease, *n* (%)	5 (25%)	3 (15%)	0.695
Heart failure, *n* (%)	3 (15%)	6 (30%)	0.451
TIA/Stroke, *n* (%)	2 (10%)	3 (15%)	1.000
OSAS, *n* (%)	0 (0%)	3 (15%)	0.231
DOAC, *n* (%)	19 (95%)	19 (95%)	1.000
Class I/III AAD at baseline, *n* (%)	4 (20%)	3 (15%)	1.000
LVEF (%)	55.0 (51.3–55.8)	55.0 (52.0–57.0)	0.402
LAVI (mL/m^2^)	42 ± 16	36 ± 14	0.392
CHA_2_DS_2_-VA score	2.5 (1.25–4.0)	4.0 (3–5)	0.639
BMI (kg/m^2^)	28.5 ± 5.1	26.8 ± 4.6	0.450

Values are presented as median [interquartile range], mean ± standard deviation or number (percentage), as appropriate. AF = atrial fibrillation; AAD, Antiarrhythmic drug; DOAC, Direct oral anticoagulation; LAVI, Left atrial volume index; LVEF, Left ventricular ejection fraction; OSAS, Obstructive sleep apnea syndrome; TIA, Transient ischemic attack.

### Procedural characteristics

Acute PVI was achieved in all patients (100%). Left atrial dwell time and procedure duration were significantly longer with BiB compared to PS (46.0 vs. 23.0 min, *p* < 0.001) and (62.5 vs. 37.5 min, *p* < 0.001). Contrast volume was higher in the BiB group (45.0 vs. 30.0 mL, *p* < 0.001), whereas fluoroscopy time was similar between systems (*p* = 0.068). PS required a substantially greater number of applications across all PV (median total 32.0 vs. 16 *p* < 0.001). No major acute complications were observed (Table 2).

**Table 2:** Procedural characteristics of the study population.

Variable	BiB Group (*n* = 20)	PS Group (*n* = 20)	*p*-value
Dose Area Product (Gy·cm^2^)	335.0 (220.5–623.5)	427.5 (291.5–628.8)	0.373
Fluoroscopy time (min)	7.10 (5.20–9.00)	8.75 (7.13–10.38)	0.068
Successful acute PVI, *n* (%)	20 (100)	20 (100)	1.000
First-pass PVI, *n* (%)	20 (100)	20 (100)	1.000
Left atrial dwell time (min)	46.0 (30.0–63.0)	23.0 (18.0–28.0)	<0.001
Procedure duration (min)	62.5 (40.0–73.5)	37.5 (34.25–44.50)	<0.001
Cardioversions (n)	0.0 (0.0–1.0)	0 (0–1.0)	0.161
Contrast amount (mL)	45.0 (40.0–50.0)	30.0 (26.25–37.5)	<0.001
Intraprocedural complications (*n*)	0 (0)	0 (0)	1.000
LSPV applications (*n*)	4.0 (4.0–4.0)	8.0 (8.0–9.0)	<0.001
LIPV applications (*n*)	4.0 (3.0–4.0)	8.0 (8.0–10.0)	<0.001
RIPV applications (*n*)	4.0 (3.0–4.75)	8.0 (8.0–9.0)	<0.001
RSPV applications (*n*)	3.5 (2.0–4.0)	8.0 (8.0–8.75)	<0.001
Total number of applications (*n*)	16.0 (13.0–17.0)	32.0 (32.0–36.0)	<0.001

Values are presented as median [interquartile range], mean ± standard deviation or number (percentage), as appropriate. LSPV, Left superior pulmonary vein; LIPV, Left inferior pulmonary vein; RSPV, Right superior pulmonary vein; RIPV, Right inferior pulmonary vein.

### Biomarkers—pre/post comparisons

In the BiB group, significant increases were observed in leukocytes, CRP, bilirubin, LDH, myoglobin, CK, and troponin, accompanied by a decrease in hemoglobin, a mild but significant reduction in haptoglobin. Renal markers, including creatinine and GFR, remained stable. In the PS group, comparable significant elevations in inflammatory and hemolytic biomarkers, as well as biochemical myocardial injury were observed, together with a more pronounced decrease in haptoglobin. Renal function was stable overall, with one PS patient showing a marked postprocedural creatinine rise and GFR drop (KDIGO stage 2 AKI), visible as an outlier in the box-plot analysis (Table 3).

**Table 3:** Comparison of pre- and post-procedural biomarker values in the balloon-in-basket and pulseSelect PFA-PVI groups.

Parameter	Timing	BiB Group (*n* = 20)	*p*-value	PS Group (*n* = 20)	*p*-value
Leukocytes (×10^9^/L)	Pre	6.34 ± 1.55	0.002	6.02 ± 1.96	0.010
	Post	8.27 ± 2.46		7.57 ± 2.38	
Hemoglobin (g/dL)	Pre	14.06 ± 1.6	<0.001	12.82 ± 1.64	0.113
	Post	13.28 ± 1.27		12.52 ± 1.67	
Platelets (×10^9^/L)	Pre	194.0 (170.5–225.3)	0.021	168.0 ± 55.4	0.694
	Post	181.0 (154–232.0)		170.3 ± 53.7	
CRP (mg/L)	Pre	1.49 (1.28–5.44)	<0.001	1.55 (0.94–3.49)	<0.001
	Post	6.77 (5.79–9.46)		5.85 (3.34–11.97)	
Haptoglobin (mg/dL)	Pre	117 ± 73	0.034	75 (55–126)	0.011
	Post	108 ± 73		63 (43–126)	
Total Bilirubin (µmol/L)	Pre	10.7 (7.2–12.4)	0.009	11.85 (8.90–15.70)	0.015
	Post	13.1 (7.2–18.0)		15.05 (10.43–25.15)	
LDH (U/L)	Pre	174.0 (164.0–236.0)	<0.001	202.9 ± 34.3	<0.001
	Post	227.0 (208.0–257.0)		253.6 ± 55.9	
Creatinine (µmol/L)	Pre	91.8 ± 17.6	0.274	101.9 (91.0–112.8)	0.776
	Post	96.2 ± 27.0		100.5 (87.2–118.8)	
GFR (mL/min)	Pre	71.3 ± 12.6	0.323	54.5 (50.0–60.8)	0.410
	Post	68.6 ± 15.1		52.0 (48.0–63.0)	
Myoglobin (µg/L)	Pre	42.0 (36.0–50.0)	0.002	69.0 (38.0–79.8)	0.024
	Post	53.0 (46.0–75.0)		75.0 (47.0–93.3)	
CK (U/L)	Pre	109.0 (62.0–189.0)	<0.001	91.5 (41.8–128.0)	<0.001
	Post	279.0 (203.0–447.0)		214.5 (140.3–307.8)	
Troponin (ng/L)	Pre	8.0 (5.0–12)	<0.001	16.3 (6.6–19.3)	<0.001
	Post	1,006.0 (699.0–1,402.0)		1,289.0 (625.5–1,873.3)	

Values are presented as median [interquartile range] or mean ± standard deviation. CK, Creatine kinase; CRP, C-reactive protein; eGFR, Estimated glomerular filtration rate.

### Intergroup comparison of delta values

**Figure 2:**
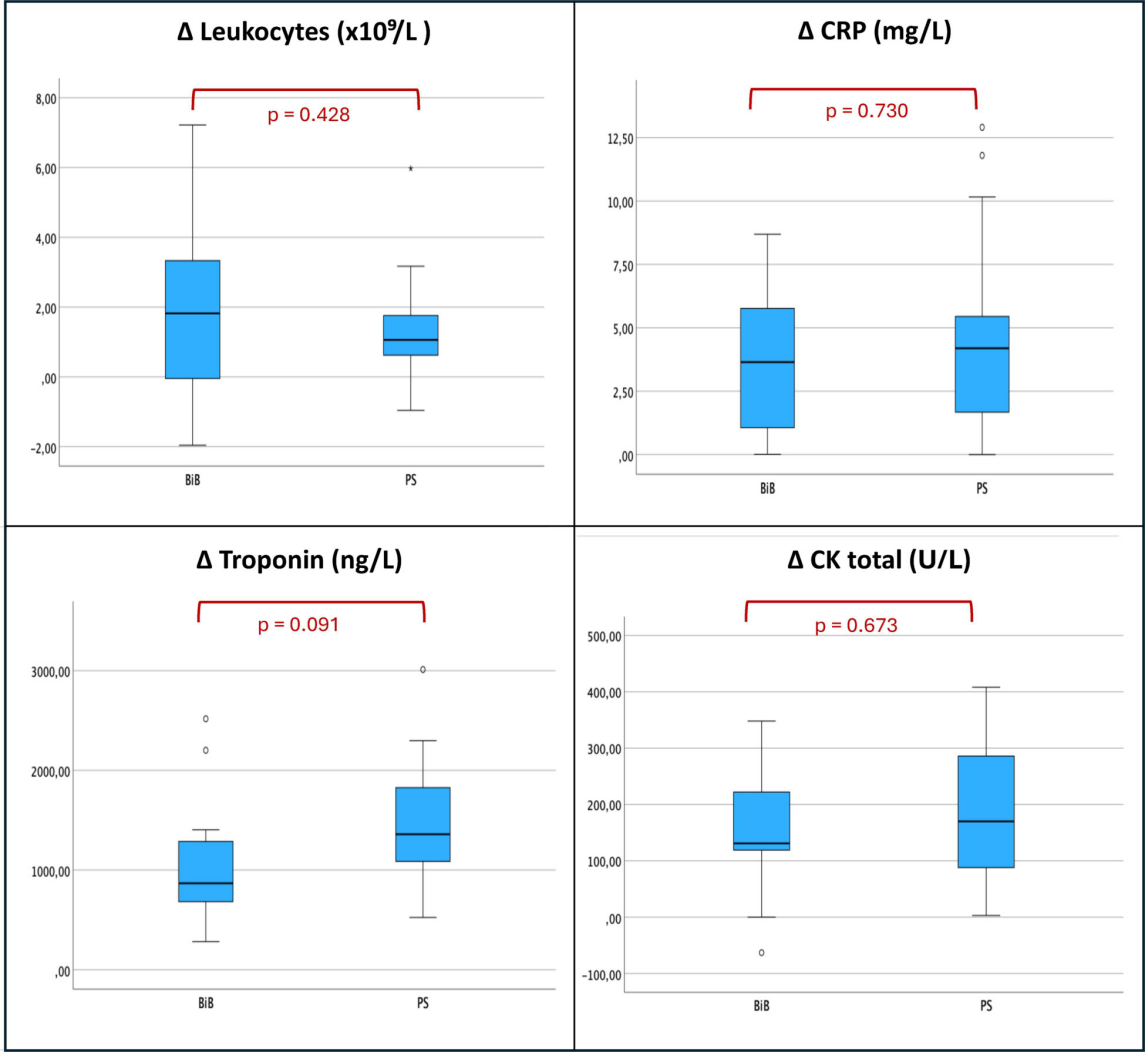
Comparison of delta values between the balloon-in-basket PFA-PVI group and the pulseSelect PFA-PVI group. Boxplots represent the median with interquartile ranges (IQR) of changes from baseline to 16–18 h after the procedure. *CRP*, *C-reactive protein,* CK, creatine kinase.

**Figure 3:**
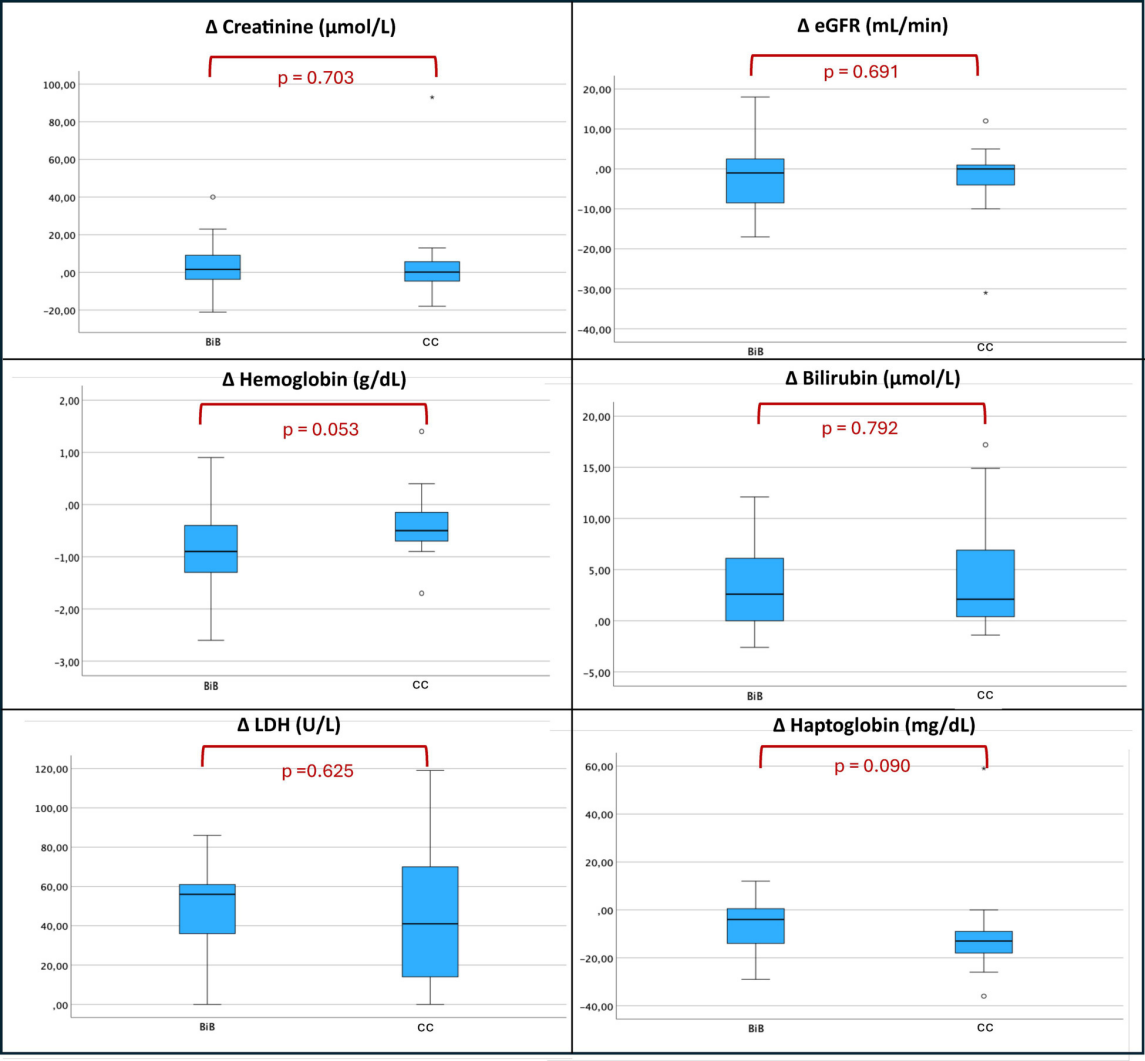
Comparison of delta values between the balloon-in-basket PFA-PVI group and the pulseSelect PFA-PVI group. Boxplots represent the median with interquartile ranges (IQR) of changes from baseline to 16–18 h after the procedure. LDH, Lactate dehydrogenase, eGFR, Estimated glomerular filtration rate.

Between-group comparisons revealed no statistically significant differences in Δ-values (Table 4, Figures 2 and 3). However, Haptoglobin showed a numerically greater reduction with PS than with BiB (Δ −13 vs. −4 mg/dL; *p* = 0.090), while hemoglobin decreased slightly more in the BiB group compared with PS (Δ −0.90 vs. −0.50 g/dL; *p* = 0.053). Changes in all remaining biomarkers—including leukocytes, CRP, bilirubin, myoglobin, LDH, creatinine, GFR, CK, and troponin—were similar between groups (all *p* > 0.05).

**Table 4:** Comparison of periprocedural biomarker changes (delta values) between the balloon-in-basket and pulseSelect PFA-PVI groups.

Marker	BiB Group (*n* = 20)	PS Group (*n* = 20)	*p*-value
Δ Leukocytes (10^9^/L)	1.82 (−0.34–3.43)	1.06 (0.50–1.92)	0.428
Δ Haptoglobin (mg/dL)	−4 (−14–1)	–13 (−19–−9)	0.090
Δ Myoglobin (ng/mL)	11.50 (4.75–46.50)	11.5 (2.25–23.5)	0.647
Δ Hemoglobin (g/dL)	−0.90 (−1.35–−0.40)	−0.5 (−0.7–0.32)	0.053
Δ CRP (mg/L)	3.65 (1.06–5.94)	4.19 (1.64–5.59)	0.730
Δ Bilirubin (µmol/L)	2.60 (−0.05–6.30)	2.10 (0.30–6.90)	0.792
Δ GFR (mL/min)	−1.00 (−10.00–3.00)	0.00 (–4.50–1.50)	0.691
Δ LDH (U/L)	48.6 ± 23.4	43.5 ± 35.1	0.625
Δ Creatinine (µmol/L)	1.60 (−4.00–11.20)	0.15 (−5.33–6.83)	0.703
Δ Troponin (ng/L)	867.5 (680.5–1,342.3)	1,358.7 (978.1–1,852.5)	0.091
Δ CK total (U/L)	162.1 ± 107.6	178.5 ± 117	0.673

Values are presented as mean and standard deviation or median [interquartile range]. Delta = post-procedural minus pre-procedural value. CK, Creatine kinase; CRP, C-reactive protein; eGFR, Estimated glomerular filtration rate.

### Correlation between number of PFA applications and biomarker changes

In the BiB group, no significant correlations were found between the number of applications and most biomarker changes, except for a mild inverse relationship between hemoglobin and application number (*r* = –0.450, *p* = 0.046). In the PS group, the number of applications correlated positively with troponin (*r* = 0.496, *p* = 0.036) and CK (*r* = 0.563, *p* = 0.020), whereas no significant relationships were found for other biomarkers (all *p* > 0.05; Table 5).

**Table 5:** Correlation between number of PFA impulses and biomarker changes in the balloon-in-basket and pulseSelect PFA-PVI groups.

Marker	BiB Group (*n* = 20)	*p*-value	PS Group (*n* = 20)	*p*-value
Leucocytes	*r* = −0.155	0.514	*r* = −0.066	0.801
Platelets	*r* = −0.231	0.327	*r* = −0.056	0.831
CRP	*r* = 0.375	0.152	*r* = −0.249	0.320
Creatinine	*r* = −0.037	0.881	*r* = 0.020	0.944
GFR	*r* = 0.1590	0.517	*r* = 0.213	0.413
Myoglobin	*ρ* = −0.329	0.250	*r* = −0.080	0.770
CK	*r* = 0.231	0.372	*r* = 0.563	0.020
Troponin T	*r* = 0.051	0.852	*r* = 0.496	0.036
LDH	*r* = 0.138	0.598	*r* = 0.167	0.522
Haptoglobin	*r* = −0.449	0.081	*r* = −0.269	0.297
Hemoglobin	*r* = −0.450	0.046	*r* = 0.056	0.836
Total Bilirubin	*r* = −0.438	0.079	*r* = 0.020	0.936

CK, Creatine kinase; CRP, C-reactive protein; eGFR, Estimated glomerular filtration rat*e.*

## Discussion

This prospective study compared systemic biological effects during PVI among two PFA technologies—the BiB catheter and the PS system.

The main findings are:

1. Both platforms induced significant increases in markers of inflammation, hemolysis, and biochemical myocardial injury.
2. Haptoglobin decreased in both groups, slightly non significantly more with PS, which may reflect differences in hemolytic response.
3. Both groups exhibited correlations between application number and biomarker changes, albeit with marker-specific patterns, suggesting a potential association between application number and biomarker changes.
4. Renal function was preserved overall, except for one PS patient who developed a transient KDIGO stage 2 AKI, likely multifactorial and reversible.

### Myocardial injury: comparable in magnitude, distinct in correlation patterns

Both systems were associated with significant increases in troponin, CK, and myoglobin, consistent with substantial biochemical myocardial injury and were in line with prior PFA studies (9–12). Intergroup comparisons showed no statistically significant differences, although BiB required fewer applications. Similar troponin and CK rises despite fewer BiB applications may suggest differences in per-application effectiveness. However, as lesion size and durability were not directly assessed, this observation may also reflect differences in dosing protocols and energy delivery strategies rather than intrinsic differences in lesion efficiency. Whether these biomarker elevations reflect irreversible necrosis or partly reversible electroporation remains uncertain. Their prognostic significance for long-term atrial function or arrhythmia recurrence warrants further study.

### Inflammation: comparable and modest systemic inflammatory activation

Both PFA systems induced a significant rise in leukocytes and CRP after ablation, reflecting a systemic inflammatory response. The magnitude of these changes was similar, with no statistically significant differences observed between groups, potentially indicating no relevant influence of catheter design on inflammatory burden in this study due to modest group sizes. No correlation with pulse count was observed, suggesting that the inflammatory response was not dose-dependent within the applied energy range. However, larger studies prospective multicenter studies are needed to conform these results. Overall, PFA-induced inflammation appears to be a uniform biological effect of electroporation rather than a catheter-specific phenomenon in this study. The modest and comparable response in both groups underscores the similar biocompatibility of the two catheters and aligns with prior reports that PFA causes only limited systemic inflammation compared with thermal ablation techniques (12–14).

### Hemolysis: evidence of device-specific signatures

Markers of hemolysis increased after ablation with both platforms. LDH and bilirubin rose significantly, while hemoglobin decreased modestly, reaching statistical significance only in the BiB group, though the absolute decline remained clinically irrelevant and likely reflects procedural hemodilution rather than true destruction of erythrocytes. This interpretation is further supported by a parallel reduction in platelet counts, consistent with hemodilution. Haptoglobin levels declined significantly in both cohorts, with a greater reduction in PS, which may reflects less consistent wall contact and greater blood-pool interaction at the PV ostium, whereas BiB ensures circumferential tissue apposition and lateral energy directionality, limiting exposure of circulating erythrocytes. However, as these parameters were not directly assessed, these interpretations should be considered hypothetical.

One PS patient developed a transient KDIGO stage 2 acute kidney injury, characterized by a rise in serum creatinine from 113 to 230 µmol/L and a decline in eGFR from 61 to 26 mL/min within 36 h. The patient remained hemodynamically stable. This event, occurring after 32 applications, was likely multifactorial. Pre-existing mild renal impairment (baseline creatinine 113 µmol/L, eGFR 61 mL/min), chronic RAAS blockade with candesartan and spironolactone leading to a reduced renal autoregulatory reserve, and moderate direct hyperbilirubinemia at baseline (total/direct 25.5/16.1 µmol/L) in the absence of transaminase elevation likely contributed to a pre-existing pigment load and enhanced susceptibility to transient tubular stress. The post-interventional increase in hemolysis further amplified this effect. Post-procedure bilirubinemia appeared as an outlier in the bilirubin box plots. Renal function improved the following day, indicating a reversible, multifactorial process involving pigment- and volume-related tubular stress rather than structural renal injury. Apart from this case, renal function remained stable in all patients, consistent with prior studies showing PFA-related hemolysis to be largely subclinical (15–17).

Catheter design features may contribute to these differences. However, this remains speculative. BiB incorporates a semi-compliant balloon that stabilizes circumferential wall contact, with flat splines oriented laterally to direct the electric field into adjacent myocardium while shielding circulating blood. It also includes an integrated contact indicator within the electroanatomical mapping system, providing real-time feedback on tissue contact quality. This allows selective deactivation of splines directed toward the blood pool, avoiding unnecessary intravascular energy delivery. In contrast, PS deploys forward-facing electrodes without balloon support or selective deactivation, activating all electrodes simultaneously regardless of orientation, which increases the probability of off-target energy dispersion.

Application number is also relevant: hemolysis generally scales with pulse burden, but the higher counts in the PS group may reflect not only cumulative exposure but also less efficient energy transfer due to inconsistent tissue coupling. In our study, the narrow dosing protocol in the BiB cohort (≈4 per vein) restricted variability, limiting correlations, though prior multicenter datasets have confirmed a clear correlation between pulse count and hemolysis (18–20).

Waveform design may further contribute. BiB uses a biphasic waveform with lower voltage requirements due to improved tissue coupling, reducing hemolytic potential without compromising lesion efficacy. Together, these features provide a mechanistic explanation for the greater hemolytic signal observed with PS.

### Associations between application number and biomarker changes

The marker-specific correlations identified here align with broader evidence that hemolysis and biochemical myocardial injury scale with pulse burden and application count across PFA systems. The observed inverse correlation between hemoglobin change and application number in the BiB group should be interpreted with caution and may reflect procedural factors such as hemodilution rather than true hemolysis, particularly in the absence of consistent changes in other hemolysis markers. In the present study, standardized dosing—particularly the narrow range in the BiB cohort—and a modest sample size limited variability and statistical power, likely biasing correlation estimates toward the null. Larger cohorts and wider dosing ranges will likely reveal a clearer and more consistent correlation between the amount of applications and dynamic across specific biomarkers, as demonstrated in recent multicenter studies (18, 20).

### Clinical implications

Systemic biological effects of PFA may be influenced by catheter design, dosing, and patient-specific factors. While inflammation and biochemical myocardial injury appear to be generic consequences of electroporation, hemolysis was more pronounced with the PS system, likely due to less consistent wall contact. The occurrence of a transient, multifactorial KDIGO stage 2 AKI in one PS patient with pre-existing risk factors represents a single observation and should be interpreted with caution. These findings suggest that both catheter- and patient-specific characteristics should inform dosing strategies, rather than applying uniform energy delivery protocols across different PFA platforms. The data may suggest short-term feasibility and the absence of major acute biomarker safety signals. Accordingly, the observed biomarker changes should be interpreted as surrogate signals without established clinical correlation. However, given the observed differences in procedural parameters between groups, it cannot be determined whether the biomarker changes are attributable to device-specific effects or differences in procedural “dose”.

## Limitations

Several limitations must be acknowledged. First, the study was a non-randomized single-center study. The modest sample size limits statistical power and increases the risk of type II error. In addition, the risk of type I error due to multiple testing cannot be excluded, as no adjustment for multiple comparisons was applied. Accordingly, all findings should be considered exploratory. Also, device allocation was guided by availability during the study period, reflecting routine clinical practice. However, this approach inherently introduces temporal and allocation bias, which may have influenced the observed results. Second, protocol-driven dosing—especially the restricted pulse range in the BiB cohort—limited variability and may have reduced the ability to detect potential associations between application number and biomarker changes. Differences in procedural parameters between groups, including number of applications, procedure duration, and contrast volume, represent additional potential confounders, and the relative contribution of device-specific effects vs. procedural “dose” cannot be disentangled. Third, biomarker sampling was confined to the first 24 h, with the exception of one PS patient with AKI. Biomarker changes were used as surrogate endpoints without clinical or imaging-based validation. Therefore, delayed changes or recovery trajectories could not be assessed, and the clinical relevance of the observed biomarker changes remains uncertain. Fourth, a substantial proportion of BiB patients were enrolled in the VOLT^TM^ CE Mark study, potentially introducing protocol-related bias. Fifth, analysis was restricted to routine clinical markers. Additional parameters such as cytokine panels, oxidative stress markers, or urinary indices of renal tubular injury would have provided deeper mechanistic insight. In addition, although not statistically significant, the numerical imbalance in AF type between groups may represent a potential confounder. Finally, both catheters represent early-generation devices, and their systemic profiles may not fully reflect future iterations or other PFA technologies.

## Conclusion

Both BiB and PS induced systemic effects characterized by inflammation, biochemical myocardial injury, and hemolysis. Similar troponin and CK increases despite fewer BiB applications may suggest differences in per-application effectiveness. However, this observation may also reflect differences in dosing protocols and energy delivery strategies. In addition, a numerically greater, non-significant decline in haptoglobin was observed in the PS group, which may reflect differences in catheter–tissue interaction, although these interpretations remain speculative. Together with prior evidence, our findings may support a potential association between impulse count and biomarker changes. They may also suggest that both the quantity and quality of energy delivery are relevant, although this requires confirmation in larger studies.

## Data Availability

The original contributions presented in the study are included in the article/Supplementary Material, further inquiries can be directed to the corresponding author.
